# Editorial: The Emerging Role of SPECT Functional Neuroimaging in Psychiatry & Neurology

**DOI:** 10.3389/fpsyt.2022.928653

**Published:** 2022-07-04

**Authors:** Theodore A. Henderson, Philip F. Cohen, Giuseppe Cardaci, Jean-Luc C. Urbain

**Affiliations:** ^1^The International Society of Applied Neuroimaging (ISAN), Denver, CO, United States; ^2^The Synaptic Space, Inc., Denver, CO, United States; ^3^Neuro-Luminance, Inc., Denver, CO, United States; ^4^Dr. Theodore Henderson, Inc., Denver, CO, United States; ^5^Neuro-Laser Foundation, Denver, CO, United States; ^6^Nuclear Medicine, Lions Gate Hospital, Vancouver, BC, Canada; ^7^Department of Radiology, University of British Columbia, Vancouver, BC, Canada; ^8^University of Notre Dame, Fremantle, WA, Australia; ^9^Department of Radiology, Wake Forest School of Medicine, Winston-Salem, NC, United States; ^10^Department of Nuclear Medicine, Wake Forest School of Medicine, Winston-Salem, NC, United States

**Keywords:** traumatic brain injury, dementia, bipolar disorder, biomarker, ADHD, Parkinson's disease, psychiatric comorbidity, fMRI

“Emerging” is somewhat of a misnomer when used to describe the role of perfusion single photon emission computed tomography (SPECT) neuroimaging in Neurology and Psychiatry. More appropriate terms that come to mind are “misunderstood” or “ignored and undervalued”. The description which most springs to mind based on the comprehensive review by Pavel et al. (A) is “well-established, but overlooked and underutilized”. Perfusion SPECT neuroimaging has been available for a long time—over 40 years, using current tracers—still the field has not stagnated. New technology, processing techniques, normative databases, and statistical analysis algorithms have been refining the field on an ongoing basis. Amen and Easton, Pavel et al. (A) and Pavel et al. (B) provide in-depth looks at the technological advances. SPECT scans are not the Rorschach-like blobs of the 1980's, but detailed examinations of activity patterns within the human brain (using the one-off metric of perfusion), which provide a certain level of anatomical accuracy and can be compared to normative databases for differential diagnoses with high sensitivity and specificity [Pavel et al. (A)].

Yet, SPECT neuroimaging meets with sustained resistance from the fields of Neurology and Psychiatry. While the American Psychiatric Association (APA) initially embraced SPECT neuroimaging (holding seminars and workshops on its use per, Amen and Easton), now the APA and psychiatrists, in general, vilify SPECT and clinicians who utilize it (1). The untenable position of the APA (2), mired in the committee-created artificial diagnostic constructs of the Diagnostic and Statistical Manual (DSM) system (3), cannot be reconciled with the current level of neurophysiological evidence of the underpinnings of psychiatric conditions, as revealed by perfusion SPECT, quantitative electroencephalographalogram (qEEG), arterial spin echo, and functional MRI (fMRI) studies [(4, 5); Pavel et al. (A)]. Neurology as a field has taken a similar position, rejecting perfusion SPECT neuroimaging as “old”, “inaccurate”, or “experimental”. For example, they maintain that there is insufficient proof that SPECT is diagnostic or even contributory in the evaluation of traumatic brain injury (TBI). However, the sensitivity and specificity of SPECT scans in the evaluation of TBI has been studied in over 23,944 subjects [Pavel et al. (A)] and SPECT meets the criteria set forth by the American Academy of Neurology (AAN) for a Type A Recommendation based on Class II evidence from multiple large N clinical studies with control groups [Pavel et al. (A)]. Note that Class I evidence is ethically impossible in the study of TBI. Similarly, thousands of subjects have been studied using SPECT in epilepsy (over 8,500 in 10 years), and dementia (over 18,000). Perfusion SPECT is diagnostic for Alzheimer's disease with comparison to histopathology with a sensitivity of 96% and a specificity of 89% (6). Moreover, SPECT can predict the conversion from mild cognitive impairment (MCI) to Alzheimer's disease with a sensitivity of 89–97% and a specificity of 89–100%, which is arguably better than ^18^F-fluorodeoxyglucose-positron emission tomography (FDG-PET) (70–90% sensitivity/82–90% specificity) [Pavel et al. (A)].

A theme which emerged among the articles in this Research Topic was the concept of a perfusion SPECT “biomarker”. While biomarker was defined in a number of ways, the definition provided by Food and Drug Administration is rigorous: “a defined characteristic that is measured as an indicator of normal biological processes, pathogenic processes, or responses to an exposure or intervention, including therapeutic interventions.” (7). Given that biomarkers are defined as a characteristic that can indicate change, as well as a condition, ample evidence was presented for the use of perfusion SPECT neuroimaging as a biomarker. Im et al. demonstrated frontal, insular and posterior cingulate hypoperfusion in four cases of neurosyphilis. While frontal and posterior cingulate hypoperfusion are also found in Alzheimer's disease, the additional finding of insular hypoperfusion may be a unique marker. McLean et al. presented a unique “clinical pearl” of a family of five—all of whom have been diagnosed with bipolar disorder using DSM criteria. This situation provides control for many socioeconomical, genetic, and family dynamic variables. All members of this family showed similar perfusion SPECT findings—specifically increased asymmetrical perfusion of the thalamus and increased asymmetrical perfusion of the cerebral cortices—which may serve as a biomarker for bipolar disorder (McLean et al.). Amen et al. described a potential biomarker for ADHD in a large population (*N* = 1,006) defined by DSM-IV criteria, detailed clinical history, and the Structure Clinical Interview for Diagnosis (SCID) who were compared to a control group that did not meet DSM-IV criteria for any psychiatric disorder. They found that hypoperfusion in the medial anterior prefrontal (orbitofrontal) cortices, anterior cingulate gyri, bilateral temporal cortices, and cerebellar subregions 8 and 9 were highly predictive of ADHD with a sensitivity of 100% and specificity of 100% (Amen et al.). Both Alster et al. and Pavel et al. (B) described SPECT findings or biomarkers which served to differentiate forms of Parkinsonian syndromes including Progressive Supranuclear Palsy, Idiopathic Parkinson's disease, Multiple System Atrophy, and Corticobasal Syndrome. Gosset et al. examined patients with both perfusion SPECT and qEEG evidence of brain injury following head trauma. Both visual read and quantitative analysis revealed frontal lobe and temporal lobe hypoperfusion in over 90% of cases with positive qEEG findings for TBI. Indeed, perfusion SPECT found cerebral changes in 100% of cases with electroencephalographic evidence of TBI, while CT and/or MRI were negative in all but one case (Gosset et al.). This finding of a potential marker for TBI replicates and reinforces the findings described in Amen and Easton, detailed extensively in Pavel et al. (B) and identified in Best et al.. Furthermore, prior studies and reviews (4, 5, 8–14) support the use of SPECT in the evaluation of TBI.

Another aspect of biomarker utility is the demonstration of change. Perfusion SPECT scans have been utilized to demonstrate response to a number of novel treatments (15–18). Best et al. revealed two additional novel treatments effective in complex, treatment-resistant cases of depression, dementia, and TBI. They used ketamine anesthesia to facilitate more powerful transcranial magnetic stimulation than would normally be tolerated to successfully treat unipolar or bipolar depression which was unresponsive to other treatments (Best et al.). In addition, they used hyperbaric oxygen therapy combined with paraspinal injection of etanercept (a tumor necrosis factor inhibitor purported to reduce inflammation) to treat TBI and dementia. In all cases, they demonstrated improvement in clinical symptoms and neurophysiological function based on pre- and post-treatment SPECT scans. Thornton et al. illustrated the neurophysiological evidence for standard community psychiatric treatment in a large open cohort of 72 patients using pre- and post-treatment SPECT scans. These studies support the use of perfusion SPECT scans as a tool to guide treatment and improve clinical outcomes by neurophysiological assessment in psychiatric disorders. The concept that SPECT scans can guide treatment and lead to improved clinical outcome is extensively discussed by Amen and Easton, Pavel et al. (B) McLean et al., and others (4, 5, 12–14, 19–21).

One final point emerged in the articles of this Research Topic. Perfusion SPECT occupies a unique niche in neuroimaging. While much maligned for its anatomical resolution, perfusion SPECT scan offers superior temporal resolution and much higher contrast sensitivity compared to anatomical MRI, functional MRI, FDG-PET, and X-ray computed tomography (CT). Contrast is the ability to discern an abnormal signal from background. The sensitivity of CT for detecting contrast agents is in the millimolar range, while that of MRI is in the micromolar range. The sensitivity of SPECT neuroimaging for detecting a radiopharmaceutical is in the nanomolar range, exceeding MRI by a thousand-fold and exceeding CT by a million-fold. Functional MRI is an area of widespread research but suffers from low signal-to-noise ratios. Temporal resolution relates to the uptake of radiopharmaceutical. The uptake of FDG in FDG-PET imaging extends over 20 min. In contrast, the uptake of perfusion SPECT radiopharmaceuticals occurs over 40–50 s. Thus, rapid events can be captured by SPECT, including seizures, attention tasks (in ADHD), pain episodes (Bermo et al.), psychotic episodes (Kalyoncu and Gonul), and other transient events. Yet, anatomical resolution is a concern in SPECT imaging and new processing techniques [McLean et al.; Best et al.; Pavel et al. (B)] and new cadmium-zinc-telluride (CZT) camera detector technologies [Pavel et al. (B)] will lead to a dramatic improvement in anatomical resolution. Nevertheless, a Pubmed search for terms “MRI brain death” yields 4,524 references. None of them specify MRI diagnostic criteria for brain death. Now, this is partly due to the incompatibility of life support equipment with the magnetic field. Nonetheless, the MRI of a dead brain can be indistinguishable for the MRI of a live brain. Ironically, the AAN practice guidelines (22, 23) specify perfusion SPECT as a diagnostic tool to prove and support the clinical diagnosis of brain death (24). While the AAN recognizes that perfusion SPECT is more sensitive than MRI in the diagnosis of brain death, the Academy fails to recognize that perfusion SPECT is more sensitive than MRI for the more subtle brain dysfunction associated with TBI, dementia, neurotoxicity, and psychiatric illnesses. The irony is striking.

## Disclosure

TH, PC, and GC are members of the International Society of Applied Neuroimaging (ISAN), a volunteer organization devoted to the understanding and appropriate clinical utilization of SPECT brain imaging. All authors volunteered their time in the research and writing of this manuscript.

## Author Contributions

The first draft was prepared by TH. All authors contributed to the conceptualization and editing of the manuscript.

## Conflict of Interest

TH is the president and principal owner of The Synaptic Space, a neuroimaging consulting firm, CEO and Chairman of the Board of Neuro-Luminance Corporation, a medical service company, president and principal owner of Dr. Theodore Henderson, Inc, a medical service company, President of the Neuro-Laser Foundation, a non-profit organization, and member of and a former officer of the Brain Imaging Council Board of the Society of Nuclear Medicine and Molecular Imaging (SNMMI). Since 2017, TH has served in the SNMMI Brain Imaging Outreach Working Group. Currently, TH serves as president of the International Society of Applied Neuroimaging. TH has no ownership in, and receives no remuneration from, any neuroimaging company. No more than 5% of his income is derived from neuroimaging. The remaining authors declare that the research was conducted in the absence of any commercial or financial relationships that could be construed as a potential conflict of interest.

## Publisher's Note

All claims expressed in this article are solely those of the authors and do not necessarily represent those of their affiliated organizations, or those of the publisher, the editors and the reviewers. Any product that may be evaluated in this article, or claim that may be made by its manufacturer, is not guaranteed or endorsed by the publisher.

## References

[1] Tucker N. Daniel Amen is the most popular psychiatrist in America. To most researchers and scientists, that's a bad thing. Washington Post. (2012). Available online at: https://www.washingtonpost.com/lifestyle/magazine/daniel-amen-is-the-most-popular-psychiatrist-in-america-to-most-researchers-and-scientists-thats-a-very-bad-thing/2012/08/07/467ed52c-c540-11e1-8c16-5080b717c13e_story.html?noredirect=on&utm_term=.47ca50ea5098 (accessed January 21, 2019).

[2] First MB Drevets WC Carter C Dickerstein DP Kasoff L Kim KL. Clinical applications of neuroimaging in psychiatric disorders. Am J Psychiatry. (2018) 175:915–6. 10.1176/appi.ajp.2018.1750701 30173550 PMC6583905

[3] American Psychiatric Association. Diagnostic and Statistical Manual of Mental Disorders. 5th ed. Arlington VA: American Psychiatric Association. (2013). 10.1176/appi.books.9780890425596

[4] Henderson TA Uszler JM Rossiter-Thornton JF Siow Y-H Pavel DG McLean M. The American Psychiatric Association fails to recognize the value of neuroimaging in psychiatry. Interv Med Clin Imaging. (2019) 1:1–8.

[5] Henderson TA van Lierop MJ McLean M Uszler JM Thornton JF Siow YH. Functional neuroimaging in psychiatry-aiding in diagnosis and guiding treatment. What the American Psychiatric Association does not know. Front Psychiatry. (2020) 11:276. 10.3389/fpsyt.2020.00276 32351416 PMC7176045

[6] Henderson TA. The diagnosis and evaluation of dementia and mild cognitive impairment with emphasis on SPECT perfusion neuroimaging. CNS Spectr. (2012) 17:176–206. 10.1017/S1092852912000636 22929226

[7] Food Drug Administration. About Biomarkers and Qualification webpage. Available online at: https://www.fda.gov/drugs/biomarker-qualification-program/about-biomarkers-and-qualification (accessed April 3, 2022).

[8] Kapucu OL Nobili F Varrone A Booij J Vander Borght T Någren K. Procedure guideline for brain perfusion SPECT using 99mTc-labelled radiopharmaceuticals, version 2. Eur J Nucl Med Mol Imaging. (2009) 36:2093–102. 10.1007/s00259-009-1266-y 19838703

[9] Cohen PF Tarzwell R Numerow L Siow Y-H Uszler JM Pavel DG. CANM Guidelines for Brain Perfusion Single Photon Emission Computed Tomography (SPECT). Available online at: https://wwwcanm-acmnca/guidelines (accessed April 3, 2022).

[10] Stamatakis EA Wilson JT Hadley DM Wyper DJ SPECT. imaging in head injury interpreted with statistical parametric mapping. J Nucl Med. (2002) 43:476–83. 11937590

[11] Raji CA Tarzwell R Pavel D Schneider H Uszler M Thornton J. Clinical utility of SPECT neuroimaging in the diagnosis and treatment of traumatic brain injury: a systematic review. PLoS ONE. (2014) 9:e91088. 10.1371/journal.pone.0091088 24646878 PMC3960124

[12] Raji CA Willeumier K Taylor D Tarzwell R Newberg A Henderson TA. Functional neuroimaging with default mode network regions distinguishes PTSD from TBI in a military veteran population. Brain Imaging Behav. (2015) 9:527–34. 10.1007/s11682-015-9385-5 25917871 PMC4575682

[13] Amen DG Raji CA Willeumier K Taylor D Tarzwell R Newberg A. Functional neuroimaging distinguishes posttraumatic stress disorder from traumatic brain injury in focused and large community datasets. PLoS ONE. (2015) 10:e0129659. 10.1371/journal.pone.0129659 26132293 PMC4488529

[14] Henderson TA. Brain SPECT imaging in neuropsychiatric diagnosis and monitoring. EPatient. (2018) 1:40–7. Available online at: http://nmpangea.com/2018/10/09/738/

[15] Lansing K Amen DG Hanks C Rudy L. High-resolution brain SPECT imaging and eye movement desensitization and reprocessing in police officers with PTSD. J Neuropsychiatry Clin Neurosci. (2005) 17:526–32. 10.1176/jnp.17.4.526 16387993

[16] Amen DG Wu JC Taylor D Willeumier K. Reversing brain damage in former NFL players: implications for traumatic brain injury and substance abuse rehabilitation. J Psychoactive Drugs. (2011) 43:1–5. 10.1080/02791072.2011.566489 21615001

[17] Henderson TA Morries LD. SPECT perfusion imaging demonstrates improvement of traumatic brain injury with transcranial near-infrared laser phototherapy. Adv Mind Body Med. (2015) 29:27–33. 10.2147/NDT.S65809 26535475

[18] Boussi-Gross R Golan H Fishlev G Bechor Y Volkov O Bergan J. Hyperbaric oxygen therapy can improve post concussion syndrome years after mild traumatic brain injury - randomized prospective trial. PLoS ONE. (2013) 8:e79995. 10.1371/journal.pone.0079995 24260334 PMC3829860

[19] Amen DG Jourdain M Taylor DV Pigott HE Willeumier K. Multi-site six month outcome study of complex psychiatric patients evaluated with addition of brain SPECT imaging. Adv Mind Body Med Spring. (2013) 27:6–16. 23709407

[20] Amen DG Highum D Licata R Annibali JA Somner L Pigott HE. Specific ways brain SPECT imaging enhances clinical psychiatric practice. J Psychoactive Drugs. (2012) 44:96–106. 10.1080/02791072.2012.684615 22880537

[21] Thornton JF Schneider H McLean MK van Lierop MJ Tarzwell R. Improved outcomes using brain SPECT-guided treatment versus treatment-as-usual in community psychiatric outpatients: a retrospective case-control study. J Neuropsychiatry Clin Neurosci. (2014) 26:51–6. 10.1176/appi.neuropsych.12100238 24275845

[22] Practice parameters for determining brain death in adults (summary statement). The Quality Standards Subcommittee of the American Academy of Neurology. Neurology. (1995) 45:1012–4. 10.1212/WNL.45.5.1012 7746374

[23] American Academy of Neurology. Policy & Guidelines. Update: Determining Bran Death in Adults. Available online at: https://www.aan.com/Guidelines/home/GuidelineDetail/431 (accessed April 17, 2022).

[24] Donohoe KJ Agrawal G Frey KA Gerbaudo VH Mariani G Nagel JS. SNM practice guideline for brain death scintigraphy 20. J Nuc Med Tech. (2012) 40:198–203. 10.2967/jnmt.112.105130 22743146

